# Bone Marrow Aspiration and Biopsy: A Guideline for Procedural Training and Competency Assessment

**Published:** 2012-07-01

**Authors:** Katrina Jackson, Andrew Guinigundo, David Waterhouse

**Affiliations:** From Barbara Ann Karmanos Cancer Institute, Detroit, Michigan (K.J.), and Oncology Hematology Care, Cincinnati, Ohio (A.G. and D.W.)

## Abstract

The growing role of nurse practitioners (NPs) in today’s demanding health-care system has allowed for a more comprehensive and complementary approach to patient care. Within the past few years, NPs have expanded their role to include invasive procedures. Limited research in the utilization of NPs has suggested equality among procedures performed by NPs when compared with those conducted by their physician counterparts. Nurse practitioners and their colleagues need to take an active role in developing protocols to train practitioners and assess their procedural competency. We suggest such a guideline for training NPs to perform invasive procedures and to confirm procedural competency, using bone marrow biopsies and aspirates as an example. Future research should be directed not only at the overall quality of biopsies obtained, but also toward patient satisfaction scores in procedures performed by NPs.

Nurse practitioners (NPs) have been providing comprehensive and complementary care for over 40 years. In the past 10 to 15 years, however, the role of the NP has expanded to include greater responsibilities and a more extensive scope of practice. The expanded role for NPs is primarily the result of two factors: the shortage of physicians and the demand for a more cost-effective health-care system. Increasingly, NPs are being asked to assist with and perform invasive medical procedures. This allows physicians to reach a larger number of patients while NPs perform these more time-consuming procedures. The utilization of NPs for invasive procedures can also be very cost-effective with similar reimbursement for procedures performed.(Although this article focuses on nurse practitioners in particular, other advanced practitioners, such as physician asssistants and clinical nurse specialists, are increasingly expanding to a more extensive scope of practice. For such procedures as those discussed here, the fundamentals of procedural training and competency assessment can be applied for those clinicians as well.)

This article is intended to specifically outline a methodology for training and assessing competency in performing invasive procedures, using bone marrow aspirates and biopsies as an example. This methodology was developed in response to a community hospital’s refusal of privileges for NPs to perform bone marrow biopsies and aspirates at their institution. The hospital argued that there was no method to prove an NP’s competency in performing the invasive procedure, and that they would not allow credentialing until proficiency was documented.

There have been several studies suggesting that a standardized approach with a formalized training program is the most effective method in training medical/surgical procedures. In a recent study published in *The Medical Teacher, *medical residents completing a “blended approach” training program for bedside invasive procedures reported a statistically significant increase in competency after completion of the program, which was evaluated by pre- and posttest competency scores (Lenchus et al., 2011).

A similar study in 2008 examined self-perceived knowledge of medical students before and after a standardized training program and their reported comfort level with the surgical procedure, showing an overwhelming improvement after completion of the developed program (Lenhard, Moallem, Morrie, Becker, & Garland, 2008). Studies support the development of a standardized training program to adequately train for invasive procedures and later test for competency, which is used at this institution to develop a framework for training NPs in performing bone marrow biopsies. This training framework can be further adapted to the instruction of other procedures.

## Background

In 1965, the first NPs were trained at the University of Colorado. Similar programs developed across the United States shortly thereafter, and today there are approximately 140,000 practicing NPs in the United States (American Academy of Nurse Practitioners, 2010). The development and acceptance of NPs have been largely in response to the increasing shortage of physicians, dating back to the mid-1960s. The NP was initially seen as an alternative provider who functioned directly under the supervision of a physician in order to extend that physician’s practice. Since then, their role has evolved not simply to function as an alternative provider, but also to complement physician care. NPs are qualified to meet the widespread needs of most patients, focusing on preventative health and the overall well-being of their patients. Various practice areas include primary care, specialties, and subspecialties, as well as acute care and emergency medicine.

The Association of American Medical Colleges (2007) estimates an 81% increase in the number of people living with and diagnosed with cancer by the year 2020. The American Society of Clinical Oncology (2005) has reported a projection of a serious shortage of oncologists by 2020. These factors support the increased utilization of NPs. Maximizing the use of NPs with specialty- specific diagnostic and therapeutic skills that are within their scope of practice will help to alleviate the projected health-care provider shortages. With this advancement, however, a few topics surrounding patient care will no doubt need to be addressed: First, can NPs perform specialty- specific invasive procedures with the same proficiency as physicians? Second, what is the optimal way to train NPs and later determine competency of these clinical skills?

## Growing Evidence to Support NP Independence

Several studies have demonstrated NPs’ ability to care for patients in a comparable fashion to physicians. Mary Mundinger of Columbia University conducted a study published in the *Journal of the American Medical Association* in 2000 that showed equivalent patient outcomes in patients randomly assigned to NPs and physicians (Mundinger, 2000; Landro, 2008). This study mainly evaluated patients in a primary care setting looking at preventative medicine and overall comprehensive care. Similarly, a Cochrane Review of 25 articles that examined 16 studies in primary health-care services suggests that NPs can produce a high quality of care while achieving identical health outcomes for patients when compared with physicians (Laurant et al., 2009). In fact, several studies suggest patient satisfaction and overall quality of care can be superior at times with NPs (Laurant et al., 2009). The intent of NPs has never been to replace physicians but rather to provide comprehensive and complementary care to patients *in collaboration with* physicians. To better achieve this goal, there is a need and desire for NPs to expand into the role of performing invasive procedures.

## Bone Marrow Biopsies

Bone marrow aspirations and biopsies are needed for diagnosis, classification, and treatment of several different malignancies and hematologic disorders, as well as for follow-up and restaging after treatment is complete. For example, in lymphoma, a bone marrow biopsy is initially done to determine the extent of disease. Similarly, in acute and chronic leukemia, a bone marrow examination is performed before and again after treatment in order to determine patient response to treatment and confirm a patient’s possible remission (Trewhitt, 2001). The procedure involves local anesthesia followed by removing an aspirate of bone marrow fluid as well as a core or solid portion of the bone. The bone marrow aspirate and biopsy are then sent to the laboratory for pathologist review and cytogenic, immunophenotypic, and molecular analysis, along with flow cytometry.

Nurse practitioners can play a key role in obtaining these samples as well as supporting the patient before, during, and after the bone marrow biopsy and aspiration. They are able to provide necessary teaching and reassurance, enhancing the patients’ understanding of the diagnosis and treatment options. Although limited research suggests NPs can achieve results comparable to those of physicians, there is no consensus on how best to train NPs for this advancement and later to determine procedural competency (Kelly, Crotty, Perera, & Dowling, 2010; Lawson, Aston, Baker, Fegan, & Milligan, 1999).

## Scope of Practice

Nurse practitioners are subject to the rules and regulations set forth by their state licensing boards. These guidelines must be strictly followed in order for NPs to remain licensed and in good standing. The Ohio Board of Nursing, for example, utilizes a decision tree as guidance for determining whether or not the procedure is within the NP’s scope of practice (Ohio Board of Nursing, 2010).

Additional structure governing the scope of practice of NPs comes from both collaborative agreements with physicians as well as policies set forth by organizations in which the NP performs the invasive procedure. Any binding clinical documents that are required will be determined by each state. For example, in Ohio, the invasive procedure needs to be addressed in the Standard Care Agreement between the NP and collaborating physicians. Each state regulates invasive procedures differently, and it is therefore important for NPs to consult their state licensing boards prior to initiating training. Once it is determined that the procedure is within the NP’s scope of practice and all requirements are met, formal training can begin. Unlike the regulation of an NP’s scope of practice, however, individual state licensing boards typically do not regulate the training or the measures used to determine procedural competency.

## Invasive Procedures Performed by NPs

One of the few published studies available for review on NPs performing bone marrow biopsies was conducted in Ireland. This study randomly examined 30 of 156 bone marrow biopsy and aspiration pathology reports performed by NPs for an overall quality review. Results of the study demonstrated that 100% of the bone marrows performed by NPs were sufficient for diagnosis (Kelly et al., 2010). A similar study done in Birmingham, United Kingdom, yielded comparable results, concluding that with a “structured educational training program,” it is possible for NPs to obtain bone marrow specimens with satisfactory quality equivalent to those done by physicians (Lawson et al., 1999). The training program utilized in this study was not published but was noted to include the following set competencies to ensure proper instruction: “1. Anatomy of the pelvis and physiology of blood formation, 2. Implications and responsibilities of bone marrow procedures, 3. Selection of an appropriate biopsy site, and 4. Preparation of slides” (Lawson et al., 1999, p. 154).

Both studies looked at the quality of the specimen and the ability to determine a diagnosis, as well as the length of the trephine biopsy. There was very little, if any, difference reported in the specimens obtained by NPs and those obtained by physicians. Limited research evaluating other invasive procedures has yielded similar results, thus reaffirming that if the procedure is within the NP’s scope of practice and adequate training has been done, there should be no measurable difference in the results of a procedure performed by an NP and one done by physician (Gibbons & Bourne, 2009).

## Developing a Structured Training Program and Determining Competency

With each invasive procedure to be performed by an NP, a method for training and evaluation should be developed and followed to assure competency and allow for continued proficiency. There is limited literature or research guiding this development. An informal survey of several Midwest hematology/oncology program directors suggests that the training of bone marrow biopsies and aspirates typically requires written, verbal, and/or video instruction, followed by observation and eventual participation with direct supervision (D. Waterhouse, personal communication, 2011). Historically, hematology/oncology fellows often logged procedures for future demonstration of numbers performed, but there was not actual quality review of procedures and specimens obtained. Given this limited framework, we attempted to formalize both the training of bone marrow procedures as well as competency assessment.

## Step 1: Written, Verbal, and Video Instruction

Training should begin with written, verbal, and video instruction. This formal instruction should be standardized, as there are several different variations in the way bone marrow biopsies can be performed as well as numerous Internet-based instructions, with varying credibility. Written and video instruction should include the rationale for doing the bone marrow biopsy and aspirate, risks and benefits of the procedure, anatomy and physiology of biopsy sites and anatomic markings, equipment used to obtain a sample, the procedure itself, and the treatment implications of the findings.

Subscribers to *The New England Journal of Medicine* can access a video entitled “Bone Marrow Aspiration and Biopsy,” which is one example of a credible, comprehensive video review (Malempati, Joshi, Lai, Braner, & Tegtmeyer, 2010). An example of adequate written instruction, entitled “A pathologist’s perspective on bone marrow aspiration and biopsy: I. Performing a bone marrow examination,” by Riley et al. (2004), can be found in the *Journal of Clinical Laboratory Analysis*. There are numerous additional resources that can be used, but again, this instruction should be standardized for a comprehensive review. In fact, there is even an iPhone application for bone marrow biopsies and aspirates available online through the iTunes App Store (MeisterMed, 2010).

Once a suitable review is selected, each NP will use the same written and video instruction. The NP should then be able to verbalize understanding of the bone marrow biopsy and aspirate procedure. The training program can be tailored to individual learning needs as determined by those overseeing the training.

## Step 2: Observation

The next step is to complete the observation portion of the training. This should include 10 observation-only sessions to provide a solid knowledge base of how to perform the procedure itself and to familiarize NPs with the equipment used. Viewing several different practitioners is a great way to learn the varied techniques. For example, in bone marrow biopsies and aspirates, observation should include biopsies performed with patients in both the prone and decubitus positions.

## Step 3: Direct Supervision

After the observations are complete and NPs feel comfortable with their knowledge of the procedure, bone marrow biopsies and aspirates may be performed under direct supervision. The exact number of bone marrow biopsies to be done may vary, but the limited research that has been done indicates that 10 supervised biopsies is an adequate sampling to display competency (Kelly et al., 2010). It should be noted, however, that the pathology reports need to be closely reviewed on all biopsies performed by NPs being trained and that these samples should be assessed as adequate for diagnosis.

## Step 4: Pathology Review, Patient Follow-Up, and Competency Assessment

As mentioned previously, all specimens obtained need to be reviewed in order to determine that they were adequate to yield a diagnosis. Typically, a pathology report will include a statement on the quality of the specimen, whether or not it was adequate for review, as well as the trephine length. The adequacy and quality of the specimen are determined by several factors (Riley et al., 2004):

- Presence of spicules in the aspirate
- Quality preparation of aspirate smears
- Total length of the trephine biopsy core; some consider the ideal to be 1 cm while others argue a core should be 1.5 to 3 cm
- Location and number of anatomic sites
- Gauge of the biopsy needle

Although pathology reports should be routinely reviewed for adequacy, it is especially important to determine correct technique and display competency during the training process. Direct communication with the pathologist to determine the quality of the specimens will aid in this process.

Another measure to assess overall technique includes patient follow-up and report of complications. This can be achieved by having the NP call each patient 1 day after the procedure and again in 1 week to assess for any adverse events. This information should be documented. Documentation is essential and will provide the necessary information required to obtain hospital/insurer privileges. In addition to keeping a procedure log (see Table 1), the practitioner should provide a detailed procedure note that includes the following (this documentation should be signed by the supervising provider):

- Indication for the bone marrow biopsy and aspirate
- Discussion of risks and benefits
- Verification of verbal consent together with written consent
- Description of sterile technique
- Anatomical location of aspirate and biopsy
- Complications, if any
- Postbiopsy instructions and follow-up

**Table 1:**
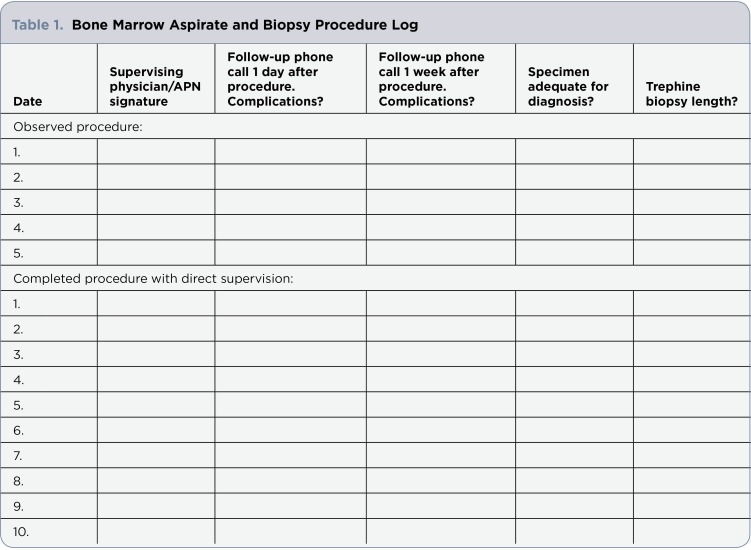
Table 1. Bone Marrow Aspirate and Biopsy Procedure Log

Continued proficiency should be demonstrated through performing the bone marrow aspirate and biopsy at least five times per year (Phelan & Latsko, 2010). If this minimum cannot be met, the collaborating physician should again sign off on the procedure after supervising the NP completing the aspirate and biopsy with 100% accuracy at least once. Overall, continued competency can be monitored through annual evaluation.

## Implications for Future Research

Specialty-specific invasive procedures are an important part of the diagnostic workup and monitoring of various medical conditions. Previous limited research in bone marrow biopsies and aspirates shows no significant difference between biopsies performed by NPs and those performed by physicians. With a growing shortage of medical professionals and a challenging health-care system, it is imperative that NPs are utilized in performing invasive procedures. Nurse practitioners and their colleagues need to take an active role in creating protocols to guide this practice and document these procedures performed. Future research should be directed not only at the overall quality of the performed procedure, but also toward patient satisfaction scores of procedures performed by NPs. This training framework can guide the development of standardized training and competency assessment in not only bone marrow biopsies and aspirates, but also in any specialty-specific invasive procedure performed by NPs.
